# Nonsurgical endodontic management of dens invaginatus: a report of two cases

**DOI:** 10.12688/f1000research.21188.1

**Published:** 2019-12-02

**Authors:** Amjad Abu Hasna, Daniela Maria de Toledo Ungaro, Allana Agnes Pereira de Melo, Karen Cristina Kazue Yui, Eduardo Galera da Silva, Frederico Canato Martinho, Ana Paula Martins Gomes

**Affiliations:** 1 Department of Restorative Dentistry, Endodontic Division, Institute of Science and Technology, São Paulo State University (UNESP), São José dos Campos, São Paulo, 12245000, Brazil; 2 Department of Science and Technology Applied to Dentistry, Institute of Science and Technology, São Paulo State University (UNESP), São José dos Campos, São Paulo, 12245000, Brazil; 3 Department of Restorative Dentistry, Operative Dentistry Division, Institute of Science and Technology, São Paulo State University (UNESP), São José dos Campos, São Paulo, 12245000, Brazil; 4 Department of Endodontics, Prosthodontics and Operative Dentistry, School of Dentistry, University of Maryland, Baltimore, Baltimore, USA

**Keywords:** Dens invaginatus, Follow-up, Root canal treatment.

## Abstract

Dens invaginatus is a malformation affecting mainly the superior lateral incisors. It is defined as an infolding of the crown hard tissues, including the enamel and dentin, and can extend up to the root apex. Root canal treatment of this abnormality is considered difficult due to the complex anatomy presented by these teeth. This case series presents nonsurgical endodontic treatment in two cases of dens invaginatus (type II and III) in maxillary lateral incisors. This nonsurgical or conventional endodontic treatment results in healing of the periapical lesions associated with both cases, with no need for extra intervention e.g. surgical or invasive management. The manual instrumentation associated with sodium hypochlorite and calcium hydroxide were able to completely heal the lesions.  Radiographic exams were carried out to control and asses the healing. Nonsurgical treatment was successful in both cases with adequate repair after a 6-year follow-up with radiographic and tomographic assessments.

## Introduction

Dens invaginatus is a growth deformity mainly affecting the superior lateral incisors. It is considered a developmental anomaly and its main characteristic is crown invagination prior to the occurrence of tooth calcification ( Rajendran & Sivapathasundharam, 2009). As it is a developmental anomaly, its pathogenetic mechanism is not yet well understood; however, the deformation is through to be due to excessive pressure during dental arch formation, internal enamel epithelium growth failure, rapid and aggressive proliferation, alteration of the enamel, tooth germ accidental fusion or infection process, or trauma injuries ( Hülsmann, 1997). In the literature, its incidence ranges from 0.04% to 10% ( Hovland & Block, 1977; Rotstein *et al.*, 1987) and the maxillary lateral incisors are considered the most affected ( Marwah *et al.*, 2009).

Dens invaginatus is grouped into three categories according to the degree of invagination. Type I characterizes an enamel invagination well delimited to the tooth crown; type II is a delimited type of enamel that surrounds the root and can interconnect with the pulp; type III is a severe condition in which invagination exceeds the limit of the cementoenamel junction generating a second foramen ( Oehlers, 1957).

The root canal system of dens invaginatus is characterized by morphologic and anatomic complexity, thus, the diagnoses and treatment planning of such cases is considered difficult ( Wayama *et al.*, 2014). Treatment approaches include the sealing of the invagination, surgical or non-surgical endodontic treatment, one-step apexification technique or tooth extraction ( Demartis *et al.*, 2009; Wayama *et al.*, 2014). This case series presents nonsurgical endodontic treatment in two cases of dens invaginatus (type II and III) in maxillary lateral incisors.

## Case 1

**Figure 1.**
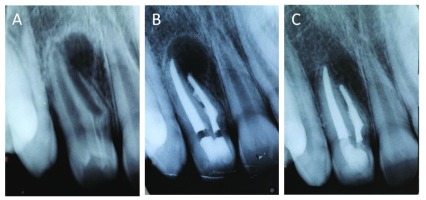
Radiographic examinations of case 1. ( **A**) Preoperative radiograph of right maxillary lateral incisor showing dens invaginatus with periapical area of radiolucency. ( **B**) Radiography after endodontic root canals filling. ( **C**) 18 months follow-up showing new bone formation in the periapical area.

A 10-year-old male was indicated to treat the right maxillary lateral incisor. Upon palpation, the patient related positive responses to percussion and palpation in the apical region. Intra-oral examination showed a small change in shape on the crown, without presence of caries or color alteration. Radiographic examination revealed dens invaginatus type III associated with periradicular lesion ( Figure 1A).

The tooth had primary and secondary canals, the secondary canal with incomplete apex and associated radiolucent area. The patient had a good general health, without anterior dental trauma history. Conventional root canal treatment was indicated.

The patient received local anesthesia (Citanest 3% - Dentsply, York, Pennsylvania, USA), absolute isolation took place, and access cavity was carried out, with two root canals being located separately without communication. Both canals presented necrotic pulp.

The canals were negotiated using size 35 K-flexofile (Dentsply-Maillefer, Ballaigues, Switzerland) and 2.5% sodium hypochlorite NaOCl (Biodinâmica, Ibiporã, PR, Brasil). The working length was measured by using Root ZX apex locator (J. Morita, Kyoto, Japan). The primary canal was instrumented through step-back technique using manual instrument, size 60 K-file (Dentsply-Maillefer, Ballaigues, Switzerland), and then the cervical third of the root canal was widened using Gates-Glidden drills. The secondary canal (invagination) was prepared with a size 90 K-file with abundant irrigation with 2.5% NaOCl.

The final irrigation of both canals was carried out with 5 mL of 17% ethylenediaminetetraacetic acid (EDTA) for 3 minutes activated by ultrasonic stream (Jet Sonic Gnatus Medical and Dental Equipment, Brazil), 5 mL of sterile saline solution to rinse out the EDTA, and sterile paper points used to dry the canals. The intra canal medication used was calcium hydroxide Ca(OH) _2_ paste (Calen, SSWhite, Rio de Janeiro, RJ, Brazil) for 14 days, the paste was spatulated over a sterile glass plate with a sterile saline solution in a proportion of 1:1 (powder: saline solution), obtaining a tooth paste-like texture

Then the patient returned for a second visit, and the tooth was asymptomatic. Clinical examination revealed no positive responses for vertical percussion or digital palpation and the soft tissues were healthy; Ca(OH) _2_ was removed. The root canals were obturated by means of thermo-mechanical compactors size 35 (Dentsply-Maillefer, Ballaigues, Switzerland) and lateral condensation technique was performed using gutta-percha cones (Tanari, Tanariman Industrial Ltda, Amazonas, Brazil) and Sealapex sealer cement (SybronEndo Kerr, Romulus Michigan, USA) ( Figure 1B). The access cavity was restored with a light cured composite resin (Z100 – 3M ESPE, Minnesota, USA).

**Figure 2.**
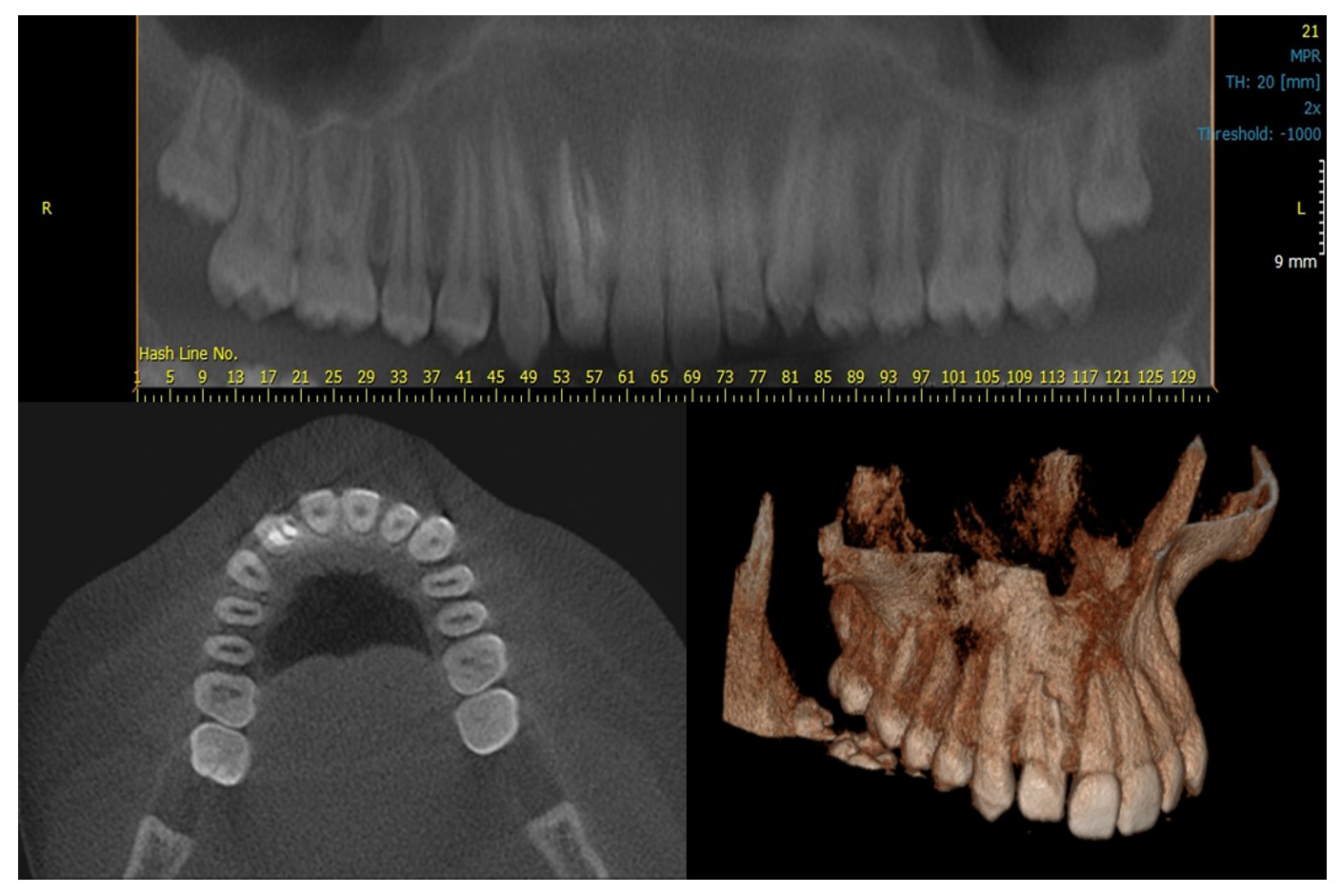
Cone beam computed tomography images 6-years after the root canal treatment in case 1.

Clinical and radiographic controls sessions were carried out at 6, 12 and 18 months after treatment ( Figure 1C). During the follow-up period, no signs and symptoms related to the respected tooth were reported by the patient. Bone neoformation was noticed in the periapical area. Figure 2 shows cone beam computed tomography 6-years after root canal treatment.

## Case 2

A 11-year-old female patient was referred for endodontic treatment, indicating sensitivity to palpation in tooth 12 and recurrent sinus tract in the vestibular area of the tooth. Endodontic treatment had been begun 8 months previously by a general practitioner, in which only one canal was located. After several unsuccessful intracanal medication changes, the practitioner referred the patient to treatment with an endodontic specialist.

**Figure 3.**
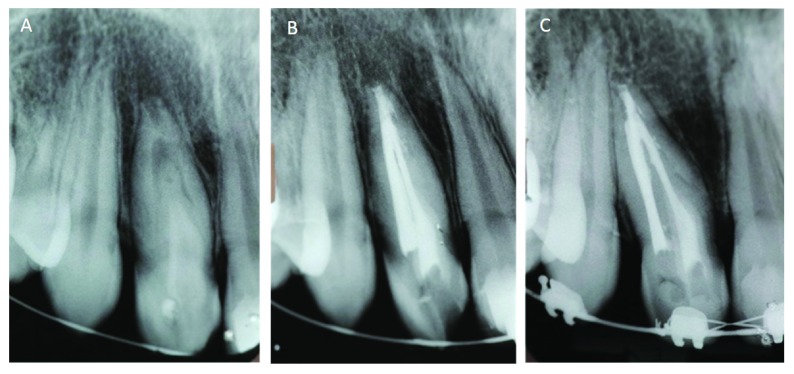
Radiographic examinations of case 2. ( **A**) Maxillary lateral incisor showing dens invaginatus and periapical lesion. ( **B**) Radiography image after root canal treatment. ( **C**) 18 months follow-up radiograph showing complete bone repair.

Radiographic examination showed the presence of dens invaginatus type II associated with periradicular lesion ( Figure 3A). A negative response to pulp sensitivity test occurred in this area.

At the first visit, after intra- and extra-oral clinical examination, the sinus tract was screened and the tooth anesthetized (Citanest 3% - Dentsply, York, Pennsylvania, USA). A rubber dam was applied, the access cavity was modified, and a second canal (invagination) was located. The root canals were negotiated with 15 K-flexofile (Dentsply-Maillefer, Ballaigues, Switzerland) and irrigated with 2.5% NaOCl.

The working length was measured using Root ZX apex locator. The primary canal was instrumented through step-back technique using manual instrument, a size 50 K-file (Dentsply-Maillefer, Ballaigues, Switzerland), and then the cervical third of the root canal was widened using Gates-Glidden drills. The second canal was prepared to a size 40 K-file using 2.5% NaOCl without use of Gates-Glidden drills.

During the instrumentation, the canals were irrigated with 2.5% NaOCl, finally rinsing with 5 mL of 17% EDTA for 3 minutes activated by ultrasonic stream. The EDTA was rinsed out using 5 mL of sterile saline solution. After drying with paper points, the canals were filled with Ca(OH) _2_ paste.

Fourteen days later, the patient related no signs and symptoms of the treated tooth and the sinus tract had cured. At this second visit, the canals were obturated with gutta-percha cones and AH Plus cement (Dentsply DeTrey GmbH, Germany) ( Figure 3B) using lateral condensation technique and the tooth was sealed with composite resin in the same session. Clinical and radiographic control of the case was performed at 6, 12 and 18 months, showing complete periapical repair ( Figure 3C). The patient underwent orthodontic treatment after the end of root canal treatment. The patient was lost to follow-up and did not come back for cone beam tomography after 6-years of the root treatment.

## Discussion

Successful endodontics treatment requires debridement and disinfection of the entire root canal system; however, complex root canal morphologic variations make the management of dens invaginatus a challenge ( Narayana *et al.*, 2012). Conventional endodontic treatment of dens invaginatus is commonly difficult and complicated, particularly when large periapical lesions are associated ( Lichota *et al.*, 2008). Nevertheless with periradicular lesion dimensions, nonsurgical endodontic treatment must be considered before any surgical treatment ( Pai *et al.*, 2004; Wayama *et al.*, 2014).

Dens invaginatus requires as early as possible diagnosis planning and treatment execution, and generally is found during radiographic investigation ( Er *et al.*, 2007). Patients often look for a professional due to acute pain or sinus tract presence. During endodontic treatment, the mechanical instrumentation of the invaginated canal might presents technical difficulties ( Lichota *et al.*, 2008).

The management of such cases when presented with necrotic pulp can be either be treatment with conventional endodontic treatment, surgical treatment, combined endodontic treatment and periapical surgery, intentional reimplantation, or tooth removal ( Beltes, 1997; Chaniotis *et al.*, 2008; Wayama *et al.*, 2014). The patient’s age and physical condition, and the case complication and variability are factors that can affect the treatment decision ( Beltes, 1997). However, type II or III dens invaginatus cases with periapical lesion, could be managed successfully through conventional endodontic treatment, and considered successful as it results in lesion regression ( Lichota *et al.*, 2008; Pai *et al.*, 2004).

In previous cases ( Alessandro *et al.*, 2018; Gound & Maixner, 2004; Nallapati, 2004; Soares *et al.*, 2017), an upper lateral incisor with type III dens invaginatus associated with periradicular lesion and vital pulp in a separate root canal have been managed non-surgically or surgically. The maintenance of pulp vitality of the main canal is possible once the invagination presents no connection with the root canal system ( de Sousa & Bramante, 1998). When one of the canals is vital but there is contact between the two canals, a pulpotomy ( Kunert *et al.*, 2017) or pulpectomy in the vital canal can be performed. In the literature, intervention in the main canal may not be necessary, especially in cases where the main canal has vital pulp or when there is no anatomical communication with the invaginated canal ( Gonçalves *et al.*, 2002).

Root canals are instrumented with manual files ( Goel *et al.*, 2017; Kumar *et al.*, 2014; Rodekirchen *et al.*, 2006; Soares *et al.*, 2017). In the present cases, the widening of the main canal with hand K-files was possible and safe but required more attention and prolonged sessions. The use of microscopy can help to define the access of the invagination and contribute in following preparations. The invagination can be cleaned and shaped with manual or rotary instruments, even if the use of rotary instrumentation within the lesion of type II dens invaginatus is not endorsed due to the fact that the surface is predominantly enclosed by enamel and has varying shapes, which may increase the possibility of endodontic instrument fracture ( Bishop & Alani, 2008).

In many cases, after the instrumentation with manual files and irrigation with NaOCl, Ca(OH) _2_ paste was primarily selected as an intracanal medication ( Gound & Maixner, 2004; Nallapati, 2004; Rodekirchen *et al.*, 2006; Soares *et al.*, 2017; Zhang & Wei, 2017). Ca(OH) _2_ is the first choice intracanal medication due to its antibacterial action ( Gound & Maixner, 2004) and dissolution of organic tissue capacity ( Hasselgren *et al.*, 1988). Ca(OH) _2_ accompanied with various vehicles can also prevent microbial growth ( Vianna *et al.*, 2005) and detoxifies residual lipopolysaccharide ( Tanomaru *et al.*, 2003). Antibiotic paste when used as an intracanal medication shows an additional effect in serious infections ( Goel *et al.*, 2017). This requires changing the paste at various times, with intervals of one month for each one and at least for 3 months ( Zhang & Wei, 2017).

Case 1 presented here was classified as dens invaginatus type III. This type of invagination allows the entrance of irritations into the periradicular area, and thus results in periapical pathology ( Gonçalves *et al.*, 2002; Jung, 2004).

Recently, pulp revascularization was introduced as a method to treat immature teeth with open apex ( Yang *et al.*, 2013). This treatment shows that apexification and total healing of the periradicular lesion occurred, showing the relevance of conservative treatment; however, surgical management may be performed in cases in which the traditional conservative management is not preferred ( Wayama *et al.*, 2014).

Computed tomographic scanning is indicated for anatomically complex cases and difficult diagnosis making ( Demartis *et al.*, 2009; Wayama *et al.*, 2014). Even more, cone beam computed tomography aids in treatment planning and execution ( Kaneko *et al.*, 2011; Patel, 2010).

Dens invaginatus associated with periapical lesion can be managed with nonsurgical endodontic treatment, which can result in acceptable periradicular curing ( Er *et al.*, 2007). The two cases presented were followed up clinically and radiographically for 18 months, indicating that healing had occurred through the bone neoformation. At 6-year follow-up, cone-beam computed tomography was performed for case 1. Both patients did not report signs, symptoms or problems with the respected teeth at follow-up visits.

### Ethical considerations and consent

These cases are part of a study assessing nonsurgical endodontic management of dens invaginatus. This study was approved by the Ethics Committee of the Institute of Science and Technology of São Paulo State University (approval number 3.711.314). Written informed consent for participation in the study was obtained from the guardians of the two patients.

Written informed consent for publication of the patients’ clinical details and clinical images was obtained from the guardians of the two patients.

## Data availability

All data underlying the results are available as part of the article and no additional source data are required.
