# Supplementary material for: Nonsurgical endodontic management of dens invaginatus: a report of two cases
Source: F1000Res. 2019 Dec 2;8:2039. [Version 1] doi: 10.12688/f1000research.21188.1 (PMC6915815; doi:10.12688/f1000research.21188.1)
Supplement: Peer review history

# Peer review: Reviewer response for version 1

**DOI:** 10.5256/f1000research.23324.r57371

**Published:** 2019-12-16

**Authors:** Vera Lúcia Schmitt

**Affiliations:** 1 Department of Dentistry, Western Paraná State University, Cascavel, Brazil

The paper is well written and can help the clinician to perform a better diagnosis and treatment plans in such cases. The case series seems to be well-executed and respected the scientific evidence in the literature.

 The abstract was concise and explained well the purpose and results obtained in the case series.

 It can be suggested the use of more recent references (2015-2019).

 In the 5th paragraph of the discussion, the authors discussed the instrumentation methods, comparing manual x rotary. It could be useful to discuss more about different points of view. 

 In the 7th paragraph of the discussion, the authors discussed new methods such as revascularization and surgical therapy. Why revascularization not used in this case? And in which cases is the traditional conservative management not preferred? This information could help the clinicians to perform better treatment planning.

I confirm that I have read this submission and believe that I have an appropriate level of expertise to confirm that it is of an acceptable scientific standard.

# Peer review: Reviewer response for version 1

**DOI:** 10.5256/f1000research.23324.r57373

**Published:** 2019-12-13

**Authors:** Carla Renata Sipert

**Affiliations:** 1 Department of Restorative Dentistry, School of Dentistry, University of São Paulo, São Paulo, Brazil

Hasna and coworkers aimed to demonstrate the success of an endodontic treatment protocol for *dens invaginatus* committed by pulpal necrosis and apical periodontitis. The authors showed a 6-year control by using cone beam computer tomography confirming the treatment success. First described by Ploquet in 1794 in a whale's tooth ^1^, this malformation was once referred to as a "tooth within a tooth". Interestingly, due to the nature of the malformation, the root canal anatomy becomes unique and unpredictable. Therefore, the clinical report here presented using conventional endodontic treatment might still be considered as the gold standard protocol to control the pulpal cavity and invagination lumen contamination.

 Overall, the manuscript is very well written. A suggestion for improving the content, the authors could include data regarding the bilateral occurrence of this anomaly at upper lateral incisors (43% ^2^) in the Introduction’s first paragraph. The structure named by the authors as “secondary canal” could be instead be named as the “invagination lumen” since structurally they are distinctly developed. The invagination lumen is not filled by pulpal tissue such as the tooth canal. Also at the sentence “Both canals presented necrotic pulp”, I would suggest to replace the term “pulp” for “necrotic tissue” as the invagination lumen is not expected to be found with pulpal tissue either vital or necrotic. Finally, when discussing the effects of calcium hydroxide, mentioning “detoxifies residual lipopolysaccharide”, please consider to cite also the study of Safavi & Nichols (1993) ^3^ who unequivocally demonstrated the LPS lipid A hydrolysis by calcium hydroxide.

I confirm that I have read this submission and believe that I have an appropriate level of expertise to confirm that it is of an acceptable scientific standard.

## References

[1] : Dens invaginatus: aetiology, classification, prevalence, diagnosis, and treatment considerations. *Int Endod J*.1997;30(2) : 10.1046/j.1365-2591.1997.00065.x 79-90 10.1046/j.1365-2591.1997.00065.x 10332241

[2] : Dens invaginatus. I. Aclinical, roentgenological and genetical study of permanent upperlateral incisors. *Odontologisk Revy*.1959;10:115-137

[3] : Effect of calcium hydroxide on bacterial lipopolysaccharide. *J Endod*.1993;19(2) : 10.1016/S0099-2399(06)81199-4 76-8 10.1016/S0099-2399(06)81199-4 8509740
